# Human Fanconi Anemia Complementation Group A Protein Stimulates the 5’ Flap Endonuclease Activity of FEN1

**DOI:** 10.1371/journal.pone.0082666

**Published:** 2013-12-04

**Authors:** Liangyue Qian, Fenghua Yuan, Paola Rodriguez-Tello, Suyog Padgaonkar, Yanbin Zhang

**Affiliations:** Department of Biochemistry & Molecular Biology, University of Miami Miller School of Medicine, Miami, Florida, United States of America; Florida International University, United States of America

## Abstract

In eukaryotic cells, Flap endonuclease 1 (FEN1) is a major structure-specific endonuclease that processes 5’ flapped structures during maturation of lagging strand DNA synthesis, long patch base excision repair, and rescue of stalled replication forks. Here we report that fanconi anemia complementation group A protein (FANCA), a protein that recognizes 5’ flap structures and is involved in DNA repair and maintenance of replication forks, constantly stimulates FEN1-mediated incision of both DNA and RNA flaps. Kinetic analyses indicate that FANCA stimulates FEN1 by increasing the turnover rate of FEN1 and altering its substrate affinity. More importantly, six pathogenic FANCA mutants are significantly less efficient than the wild-type at stimulating FEN1 endonuclease activity, implicating that regulation of FEN1 by FANCA contributes to the maintenance of genomic stability.

## Introduction

During DNA replication, various structured DNA intermediates are effectively processed to avoid deleterious consequences such as genome instability and cancer. Replication on the lagging strands is discontinuous and initiated by DNA Pol α, which synthesizes an RNA primer approximately 12-nt long that is further extended with approximately 20-nt of DNA. This RNA-DNA hybrid is believed to be made at low fidelity and subjected to displacement by the high-fidelity DNA Pol δ. The strand displacement activity of Pol δ produces a 5’ single-stranded flap structure that contains the RNA primer and some of the initiator DNA. The 5’ flap structure is then recognized and incised by the structure-specific flap endonuclease FEN1 to ensure the integrity of DNA replication [1–10]. Additionally, FEN1 has 5’ to 3’ exonuclease activity and gap-dependent endonuclease activity that are important during maturation of Okazaki fragments and the rescue of stalled replication forks [6,11–16]. FEN1 is also involved in long-patch base excision repair, nucleotide excision repair, non-homologous end-joining, and resolution of di- and tri-nucleotide repeat secondary structures [7,17–19]. Defects in FEN1 cause accumulation of mutations, genomic instability, cancer predisposition and chronic inflammation [20–22]. 

More than 30 proteins have been shown to interact with FEN1 and affect its function [7,11]. For example, proliferating cell nuclear antigen (PCNA) stimulates FEN1 endonuclease activity through protein-protein interaction [23–25]. RecQ DNA helicases such as Werner syndrome protein WRN and Bloom syndrome protein BLM were shown to stimulate FEN1 through physical interaction [26–28]. It was also reported that MUS81-EME1 and MUS81-EME2, DNA endonucleases involved in interstrand crosslink unhooking and Holliday junction resolution, stimulate FEN1 activity [29]. 

FANCA is one of the 16 Fanconi anemia disease genes [30–33]. Fanconi anemia is a severe genetic disorder characterized by bone marrow failure, genomic instability, and predisposition to cancer. Fanconi anemia cells are generally sensitive to DNA damaging agents and show hypersensitivity to DNA crosslinking compounds, indicating that they are defective in repairing or tolerating DNA damage, particularly interstrand crosslinks [30,34–42]. Deficiency of each gene shows similar clinical and cellular phenotypes; however, approximately 66% of Fanconi anemia patients presents with defective FANCA [39].

FANCA has been found to exist in a large nuclear protein complex that contains the Fanconi anemia core complex and the Bloom syndrome BLM complex [36,43]. It is localized to chromatin in a replication-dependent manner [44–46]. Xenopus egg extracts immune-depletion study shows that FANCA is directly involved in maintenance of replication forks [47,48]. Most recently, FANCA has been found to regulate MUS81-EME1 activity in a damage-dependent manner and FANCA has intrinsic affinity to nucleic acids with particularly high affinity to single-stranded RNA and DNA structures with a 5’ flap [46,49]. Because both FANCA and FEN1 localize to replication forks and practically share the same substrate specificity, i.e. 5’ flap structures and single-stranded RNA [44–46], we hypothesize that FANCA may affect FEN1 activity by competing or collaborating with each other on the same structures to regulate removal of RNA primers and 5’ flap structures during DNA replication and repair. In support of this hypothesis, deficiency in FEN1 causes partially similar phenotypes as FANCA, i.e. inflammation and cancers [22,50–52].

In this study, we found that human FANCA indeed stimulates FEN1 endonuclease activity. This novel regulation of FEN1 by FANCA is impaired in pathogenic FANCA mutants thus making the novel interaction physiologically relevant to Fanconi anemia.

## Materials and Methods

### Expression and Purification of Proteins

cDNAs for human FANCA and FEN1 were obtained by PCR amplification from a universal cDNA pool (BioChain Institute, Inc.). The full-length open reading frames were confirmed by sequencing and found to exactly match NCBI Reference Sequence NM_000135 and NM_004111 respectively. Overexpression and purification of hexahistidine-tagged FANCA was achieved in insect High Five cells using the Bac-to-Bac expression system (Invitrogen) as previous described [16,46]. Truncation mutants of FANCA were produced through a PCR-based method [53]. Point mutations were produced through a site-directed Mutagenesis Kit (Agilent). Expression of FANCA and its mutants was confirmed by Western blot analysis using FANCA Antibody (Santa Cruz Biotech.). Monoclonal antibody against the His6 tag (GenScript, Piscataway, NJ) was also used to confirm expression and subsequent purification. Protein concentration was determined using the Coomassie protein assay reagent (Pierce). The purified proteins were stored at -80 °C in aliquots. Purified FEN1 was prepared as described previously [13,54] and confirmed by western blot using a FEN1 antibody (Epitomics).

### Preparation of Substrates

Oligonucleotides that were used to create the 15-nt 5’ flap substrates were adopted from a design by Fisher et al with the same sequences (Figure S1) [55]. RNA/DNA hybrid oligos were chemically synthesized by Integrated DNA Technologies, Inc. with the flap as RNA. All DNA oligos were purified by 10% denaturing PAGE gel. The 5’ ends in the flap structures were labeled by ^32^P (Figure S1). Annealing was carried out in a water bath within 5 h by slowly cooling from 70 °C to 20 °C.

### Endonuclease Assay

The endonuclease assay was performed as previously described [1]. 2 nM of 5′ ^32^P-labeled 5’ flap substrates were incubated with purified proteins as indicated amount in a 10 μl reaction with the buffer containing 30 mM HEPES PH 7.5, 1 mM dithiothreitol, 3 mM MgCl_2_, 5% glycerol, 100 ng/mL bovine serum albumin and 100 mM KCl at 37 °C for 15 minutes. The reaction was stopped by adding 10 μl 2x sequencing dye (10 mM EDTA, 0.2% SDS, 0.03% Xylene cyanol and Bromophenol blue). Reaction products were separated on a 10% or 15% denaturing polyacrylamide gel. The incision products were visualized by autoradiography and quantified by using NIH ImageJ software. The incision rate was calculated by dividing the intensity of product band by the total substrate band of each reaction.

### Determination of Kinetic Parameters

To measure kinetic parameters, kinetic analyses were repeated three times using increasing amounts (described under the figure) 15nt both DNA and RNA 5’ flap substrates. Kinetic parameters were obtained based on the Michaelis-Menten equation: *v* = V_max_[S] / (K_m_+[S]), where *v* is the reaction rate and [S] is the concentration of substrates. K_m_ and V_max_ were gained by plotting *v* against [S] using Origin software through nonlinear curve fit. 

### Co-immunoprecipitation (Co-IP) Assay

FANCA-null (RA3087) and the FLAG-FANCA-complemented cells were generously provided by Agata Smogorzewska at the Rockefeller University [56]. Cells were grown in DMEM (sigma) with 10% FBS and harvested at 80% confluence by trypsinization. Cells were washed once by PBS and dissolved in Lysis Buffer (50 mM Tris-HCl, pH 7.6, 500 mM NaCl, 0.5 % NP-40, 2 mM EDTA, 2 mM DTT, 1x proteinase inhibitor, and 1mM Sodium orthovanadate) for sonication by using a Qsonica sonicator. Lysates were centrifuged and pre-cleaned with 10 μl activated Staph.aureus cells. 600 μg pre-cleaned extracts were incubated in buffer (50 mM Tris-HCl, pH 7.6, 100 mM NaCl, 1 mM DTT, 50 μg/mL BSA) overnight at 4 °C with the following antibodies or IgG: mouse monoclonal Anti-FLAG M2 antibody (Sigma Aldrich), rabbit polyclonal anti-FEN1 antibody (Bethyl), mouse IgG_1_ antibody and rabbit IgG antibody (Santa Cruz). Next, they were incubated with protein G magnetic beads (Millipore) for 1h at 4 °C, followed by 3 washes with ice cold Wash Buffer (50 mM Tris-HCl, pH 8.0, 5 mM EDTA, 150 mM NaCl, 0.1% NP-40, 1 mM DTT, 1x proteinase inhibitor, and 1 mM Sodium orthovanadate). 10μl of Lysis Buffer was used to elute the protein complexes from the beads and the protein complexes were resolved by 10% SDS-PAGE and transfer to nitrocellulose membranes (Bio-Rad). Blots were incubated with the following primary antibodies: goat polyclonal anti-FANCA (C-20) antibody (Santa Cruz), rabbit monoclonal anti-FEN1 antibody (for mouse FLAG co-IP, Epitomics), and mouse monoclonal anti-FEN1 (4E7) antibody (for rabbit FEN1 co-IP, Abcam), followed by incubation with HRP conjugated secondary antibodies and visualization using a Thermo Supersigal detection kit.

## Results

### FEN1 incises 5’ RNA flap differently from the DNA counterpart

**Figure 1:**
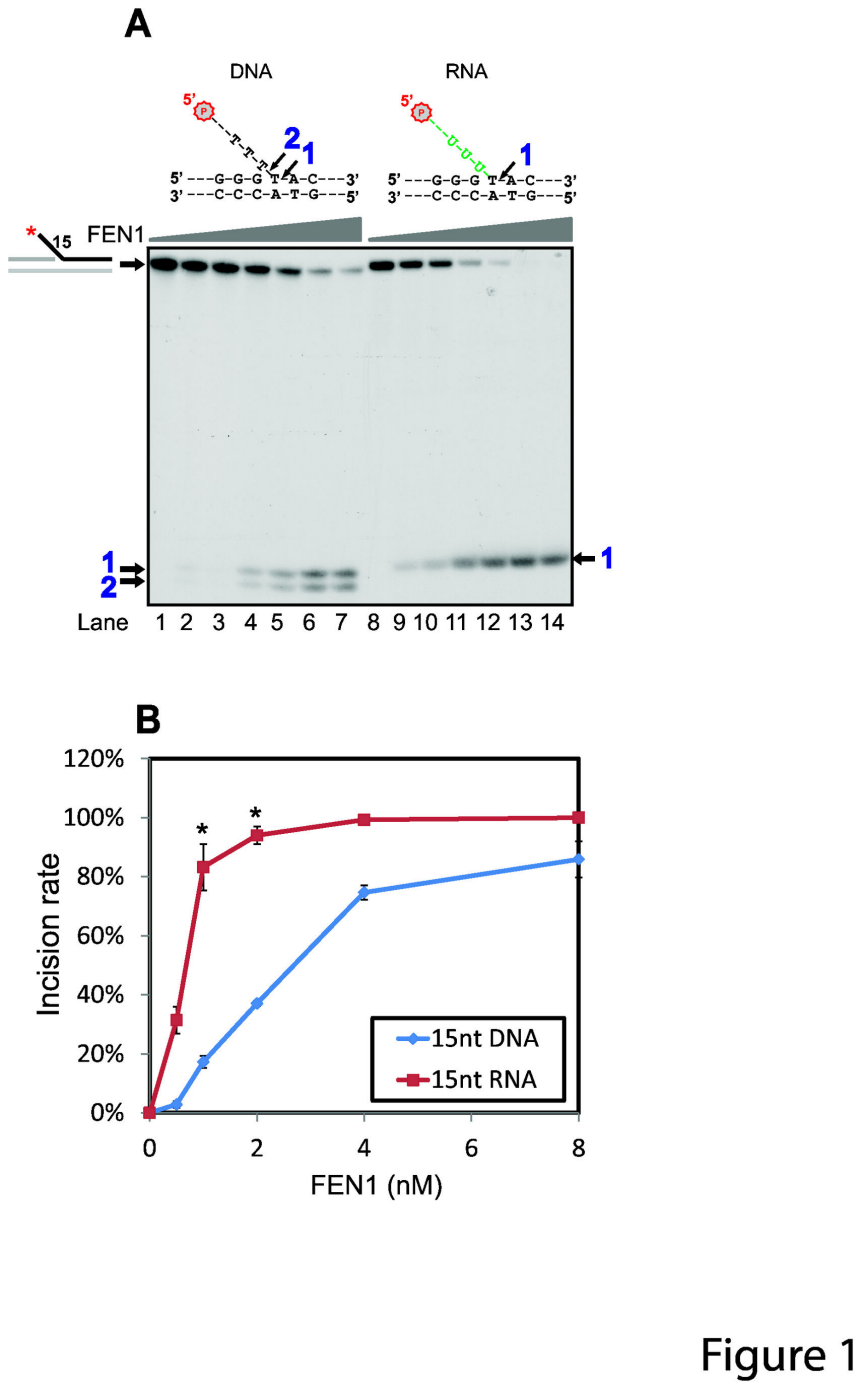
FEN1 incises 5’ RNA flap differently from the DNA counterpart. (**A**) FEN1 endonuclease assays were performed with increasing amounts of FEN1 (0.25, 0.5, 1, 2, 4, 8 nM) with both the 15-nt DNA and RNA 5’ flaps (2 nM). Diagrams of the 5’ flap substrates were shown on top of each set of reactions. Reaction products (indicated by an arrow) were resolved in 10% denaturing polyacrylamide gel. (**B**) Quantitation of FEN1 endonuclease assays in A by incision rate. Error bars represent standard deviations of three independent experiments. *, p<0.05 when compared the RNA and DNA flap reactions within the same FEN1 concentration.

In order to test whether FANCA interacts with FEN1, we overexpressed and purified full-length human FEN1 protein (Figure S1A). Considering the length of the in vivo substrate of FEN1, we designed 15-nt 5’ DNA and RNA flaps (Figure S1B). Initial incubation of the purified protein with the flap structures showed that FEN1 incised both the DNA and RNA flap structures (Figure 1A). Intriguingly, FEN1 incised the DNA flap differently from the RNA counterpart. FEN1 had two major incision sites on the 15-nt DNA flap with one right at the junction site (Figure 1A, DNA panel, arrow 2) and the other at -1 base inside the junction site (Figure 1A, DNA panel, arrow 1). However, FEN1 cut the RNA flap only at the -1 position inside the junction (Figure 1A, RNA panel). Furthermore, FEN1 incised the RNA flap significantly more efficiently than the DNA flap (Figure 1A, compare lanes 1-7 with 8-14; Figure 1B).

### FANCA stimulates the 5’ flap endonuclease activity of FEN1

**Figure 2:**
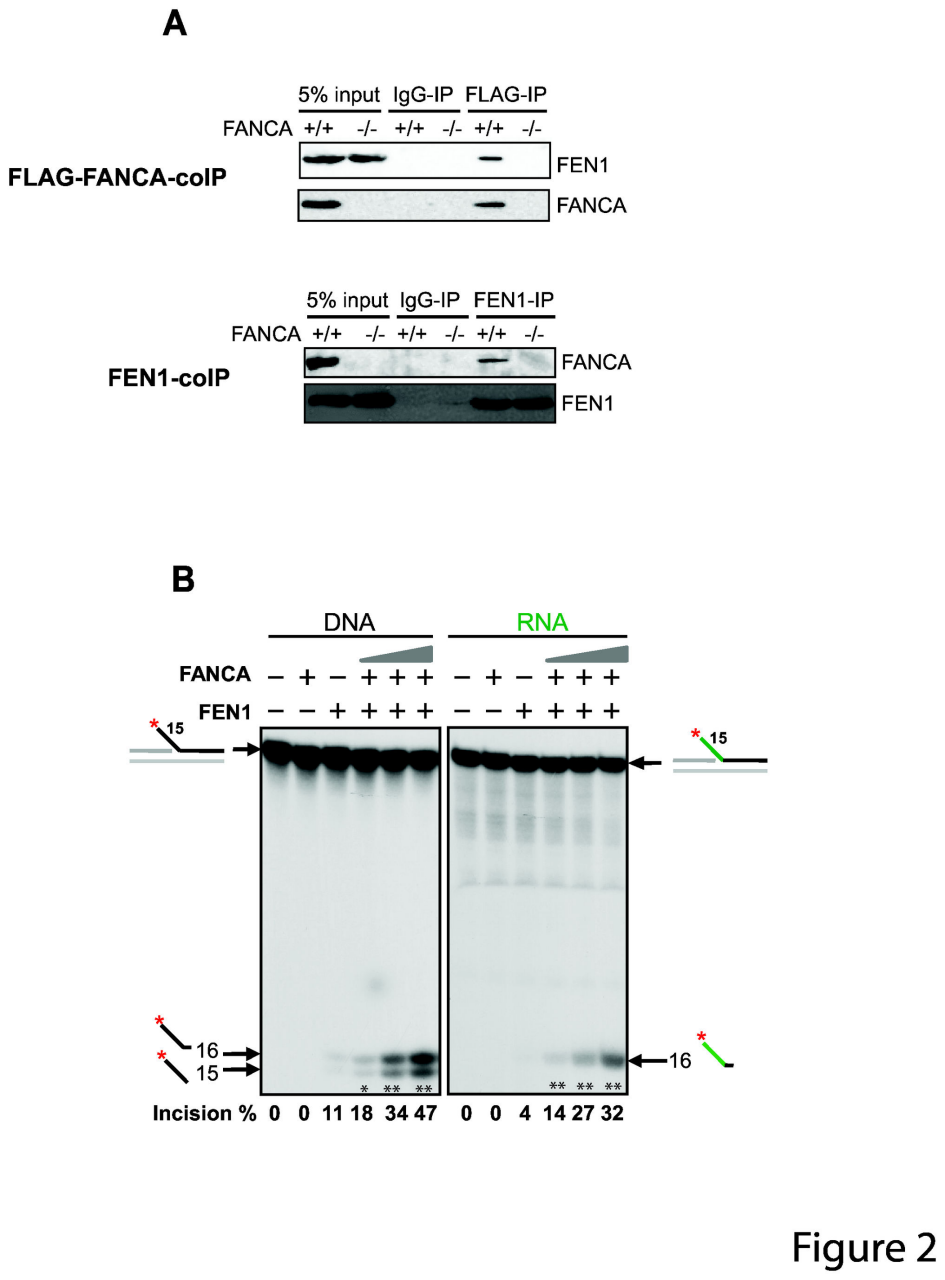
FANCA interacts with FEN1 and stimulates its incision activity. (**A**) Reciprocal co-immunoprecipitation was performed in FANCA-null (-/-) and FLAG-FANCA-complemented (+/+) cells. FLAG-FANCA was pulled down by a mouse FLAG antibody and FEN1 was pulled down by a rabbit FEN1 antibody (Bethyl). Detection of the FANCA and FEN1 was carried out by antibodies with different origins as described in Materials and Methods. (**B**) FANCA stimulates FEN1 activity. Both the DNA (black) and RNA (green) flap substrates were used at 2 nM. The concentration of FEN1 was 0.2 nM for DNA flap and 0.1 nM for RNA flap. The concentration of FANCA indicated were 2.5, 5, 10 nM. FANCA^+^/FEN1^-^ lanes: 10 nM of FANCA. Reaction products were resolved in 10% denaturing polyacrylamide gel. Arrows point to the incision sites. The incision rate is quantified and shown on the bottom. **, p<0.01; *, p<0.05 when compared to the reaction with FEN1 alone.

Since FANCA and FEN1 share the same substrate specificity, i.e. 5’ flap structures and single-stranded RNA [46], we hypothesize that FANCA physically and functionally interacts with FEN1. Physical interaction between FANCA and FEN1 has never been reported previously. In order to examine whether FANCA interacts with FEN1 in cells, we prepared whole-cell extracts of the FANCA-null and FLAG-FANCA-complemented cells (FANCA -/- and +/+ respectively in Figure 2A). Next, we performed a co-immunoprecipitation assay of the extracts using a mouse anti-FLAG antibody and detected FANCA and FEN1 using a goat anti-FANCA and a rabbit anti-FEN1 antibodies respectively (Figure 2A, top panel). As shown in Figure 2A, When FANCA was pulled down by the anti-FLAG antibody, FEN1 followed, indicating that FANCA interacts with FEN1 in cells. To confirm the physical interaction, we performed the co-immunoprecipitation assay using a FEN1 antibody (Figure 2A, bottom panel). Again, FANCA was steadily detected in the pull-down lysate. These results indicated that FANCA and FEN1 interact with each other in human cells.

To test whether FANCA functionally affects the catalytic activity of FEN1, we purified human wild-type FANCA to near homogeneity (Figure S1A). Next, we titrated the purified FANCA in a flap endonuclease assay using suboptimal amounts of FEN1 (Figure 2B, 0.2 nM for DNA flap and 0.1 nM for RNA flap respectively). To rule out the possibility of the stabilizing effect of proteins on FEN1, we diluted purified FEN1 and FANCA proteins in a buffer with 1 μg/μl BSA. Incision of the 15-nt DNA and RNA flaps by FEN1 alone is ~11% and ~4% of the total substrate respectively. However, addition of increasing amount of FANCA greatly enhanced the flap endonuclease activity of FEN1 by up to 8-fold for RNA flap and 4.5-fold for DNA flap within the titration range (Figure 2B). These data establish that FANCA physically interacts with FEN1 and functionally stimulates the flap endonuclease activity of FEN1 in a concentration-dependent manner.

### FANCA increases the enzyme efficiency of FEN1

**Figure 3:**
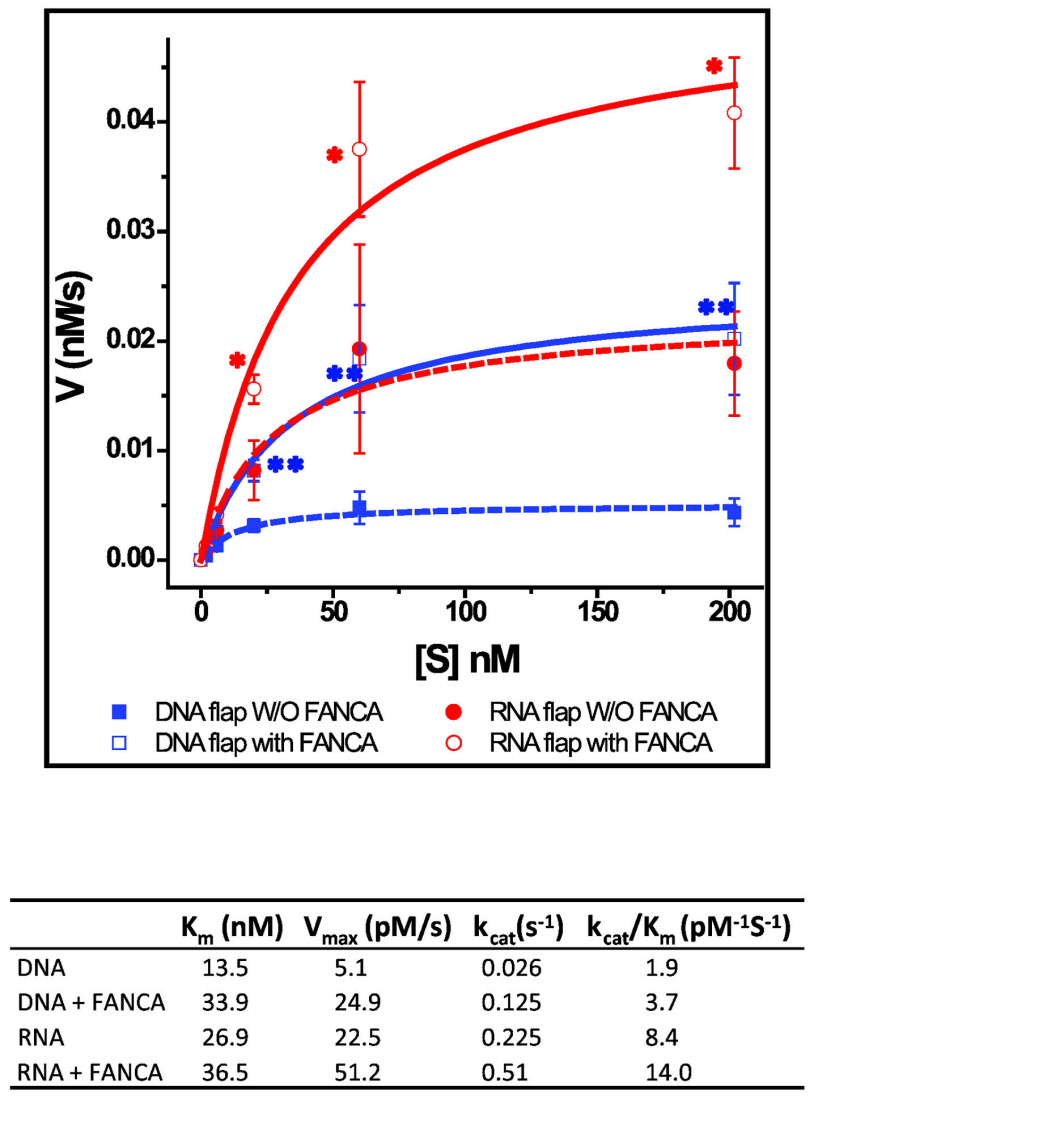
FANCA stimulates the 5’ flap endonuclease activity of FEN1 by enhancing enzyme efficiency. Steady-state analysis was perform by increasing the flap substrate concentration (0, 2, 6, 20, 60, 200 nM) and fixing amounts of FEN1 (0.2 nM for DNA flap, 0.1 nM for RNA flap) and/or FANCA (10 nM) in three independent FEN1 endonuclease assays. V_max_ is determined as described in “Material and Methods”. Error bars represent stand errors. A paired *t*-test was performed to determine the statistical significance of FEN1 endonuclease activity between with and without FANCA. **, p<0.01; *, p<0.05.

To determine how FANCA affects the flap endonuclease activity of FEN1, we performed a steady-state analysis of FEN1 by titration of the DNA and RNA flap substrates in the presence or absence of FANCA (Figure S2). The obtained incision rate (v) and the substrate concentration [S] were fit into the Michaelis-Menten equation *v* = V_max_[S] / (K_m_+[S]) in a nonlinear manner (Figure 3). As shown in Figure 3, FANCA resulted in ~4-5 fold increase in FEN1 enzyme turnover rate on the DNA flap (*k*_cat._ 0.125 vs. 0.026). However, FANCA reduced the DNA flap substrate affinity of FEN1 (K_m_ 13.5 vs. 33.9). Overall, FANCA increased the enzyme efficiency of FEN1 by 2-fold (*kcat*/K_m_ 1.9 pM^-1^s^-1^ vs. 3.7 pM^-1^s^-1^). A similar result was obtained with the RNA flap substrate (Figure 3), i.e., FANCA increased the turnover as well as K_m_ of FEN1 with a 1.7-fold overall increase in enzyme efficiency. This result is distinct from that of PCNA which decreased the K_m_ of FEN1 [25], as well as that of WRN or RFC, which increased the V_max_ but did not alter substrate binding [28,57]. 

### Both N- and C-terminals of FANCA are required for the stimulation of FEN1 activity

**Figure 4:**
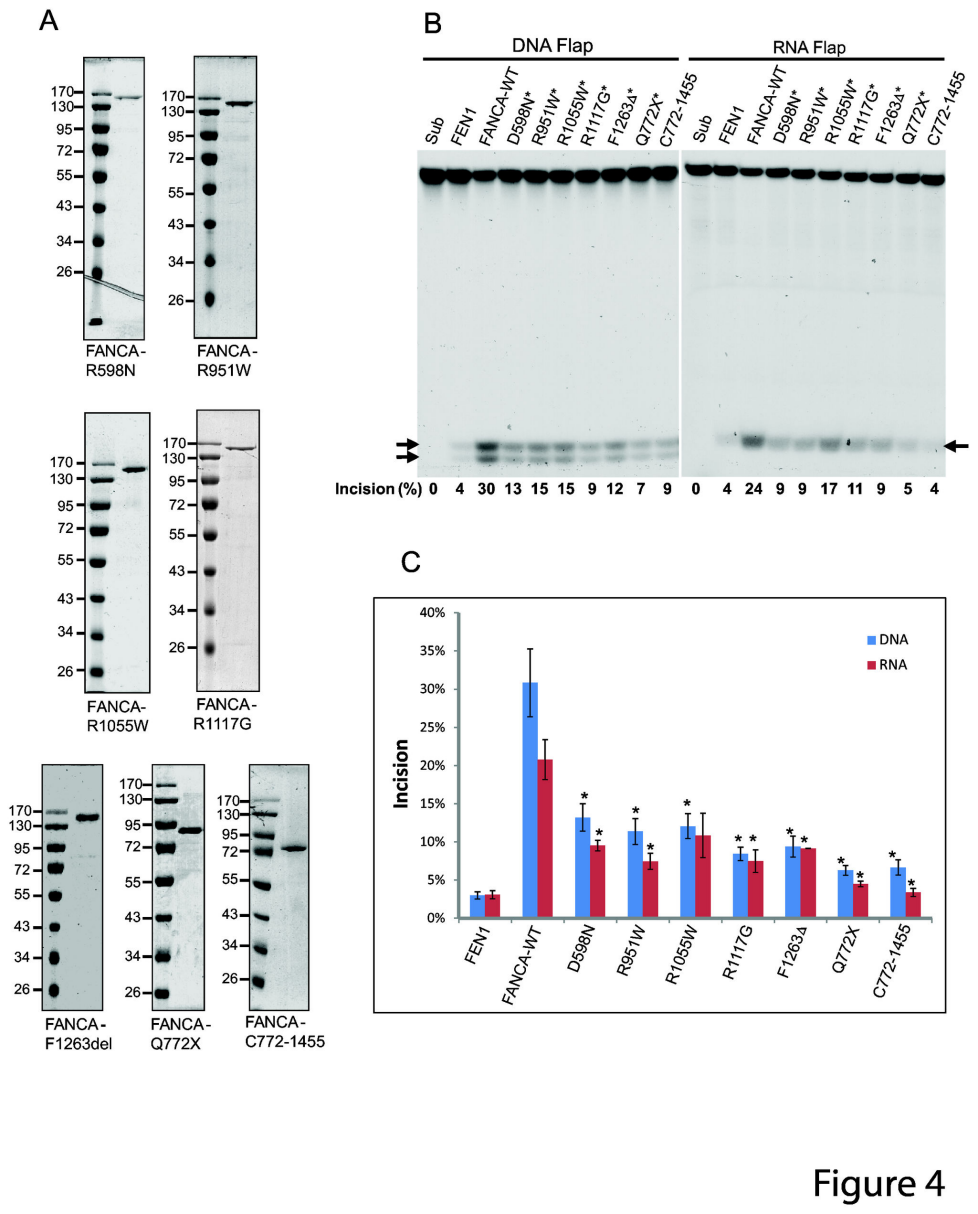
Pathogenic FANCA mutant proteins are inefficient in stimulating FEN1 endonuclease activity. (**A**) SDS-PAGE analysis of purified FANCA mutant proteins. Proteins were subjected to 10% gel electrophoresis and the gel was stained with Coomassie Brilliant Blue R-250. D598N, R951W, R1055W, R1117G, F1263del, and Q772X are pathogenic FANCA mutants. C772–1455 is C-terminal residues 772–1455 of FANCA. All peptides shown were tagged with hexahistidine at their N termini. Protein markers in kilodaltons were indicated on the left. (**B**) FEN1 endonuclease assays were performed with WT and mutant FANCA protein for both 15nt length DNA and RNA 5’ flap substrates. The concentration of FEN1 was 0.2 nM for DNA flap and 0.1 nM for RNA flap. The concentration of FANCA was 10 nM. Reaction products were resolved in 15% denaturing polyacrylamide gel. Arrows point to the incision products. The incision rate is quantified and shown on the bottom. (**C**) Quantitation of three independent FEN1 endonuclease assays in B by incision rate. Error bars represent stand errors. A paired *t*-test was performed to determine the statistical significance of FEN1 endonuclease activity between WT and mutant FANCA. *, p<0.05.

We previously showed that FANCA has a nucleic acid binding domain at the C terminal and this domain confers the preferential binding of FANCA to ssRNA, ssDNA, and 5’ flaps [58]. To test whether the nucleic acid binding domain of FANCA affects the stimulation of FEN1 by FANCA, we used two truncation mutants of FANCA, Q772X and C772-1455 for FEN1 assay. Q772X is a Fanconi anemia disease-causing C-terminal truncation mutant. C772-1455 is the complementing C-terminal fragment of Q772X (Fig. 4A). Using 10 nM of protein that is sufficient for the WT protein to exert its stimulation, we found that both mutants showed drastic reduction in stimulating FEN1 endonuclease activity (Figure 4B and 4C, last two lanes in each panel). These results indicate that both the N-terminal and the nucleic acids binding C-terminal of FANCA are indispensable for FEN1 stimulation and that the DNA binding domain by itself is insufficient to regulate FEN1 activity.

### Pathogenic FANCA mutants are significantly less efficient in stimulating FEN1 activity

To evaluate whether stimulation of FEN1 by FANCA is physiologically relevant to Fanconi anemia, we created 5 more FANCA point mutations and purified them to near homogeneity (Fig. 4A). D598N, R951W, R1055W, R1117G, and F1263Δ are selected from FANCA mutations that cause Fanconi anemia [59,60]. D598N, R951W, R1055W, and R1117G are pathogenic missense point mutations. F1263Δ is a one residual deletion mutant representing one of the most prevalent pathogenic point mutations. Using 10 nM of protein, we found that all of the FANCA disease-causing mutants have defects in stimulating the endonuclease activity of FEN1 (Figure 4B and 4C). These results clearly demonstrate that mutations in FANCA significantly affect its ability to stimulate FEN1 and the interaction between FANCA and FEN1 is relevant to the etiology of Fanconi anemia.

## Discussion

In this study, we aimed to unveil the function of the preferential 5’ flap and RNA binding activity of FANCA [46] and found a novel physical and functional interaction between FEN1 and FANCA. We demonstrated that FEN1 incises a RNA flap more efficiently than its DNA counterpart and FANCA further stimulates this incision catalyzed by FEN1. More importantly, all six pathogenic FANCA mutant proteins we tested were defective in this interaction to different degrees, indicating that the interaction between FEN1 and FANCA physiologically contributes to the pathogenesis of Fanconi anemia.

Intriguingly, before we study how FANCA may affect FEN1 activity, we observed that FEN1 alone, different from a previous study reporting that FEN1 does not cleave the 5’ RNA flap structure [61], incises the 5’ RNA flap more efficiently than its DNA counterpart (Figure 1). This is obviously beneficial to its function in RNA primer removal during maturation of Okazaki fragment. Additionally, the incision pattern for the RNA flap is different from the DNA flap (Figure 1). It showed that FEN1 only cleaves the RNA flap substrate at the -1 position inside the junction (Figure 1A, RNA panel) which is actually a DNA base pair. However, there were two cleavage sites on the DNA flap substrate: one at the junction site and the other at -1 base inside the junction site (Figure 1A, DNA panel). This is different from a previous study showed that mammalian FEN1 cuts DNA flaps only at the -1 position inside the junction site [61]. Our data supports that the nature of the 5’ flap affects the incision sites and efficiency of FEN1 initially proposed by Bambara’s group [62].

FEN1 interacts with >30 proteins and plays pivotal roles in several DNA metabolic pathways including maturation of Okazaki fragments, rescue of stalled replication forks, long-patch base excision repair, nucleotide excision repair, non-homologous end-joining, resolution of di- and tri-nucleotide structures, and apoptotic DNA fragmentation [6,7,11–19].

Besides the physical interaction we showed in Figure 2 and the shared substrate specificity of FEN1 and FANCA for 5’ flap and RNA, our confocal microscopy result showed that FANCA perfectly colocalizes with replication forks in unstressed human cells (Qian et al, unpublished data), indicating that FANCA is associated with the normal replication machinery where FEN1 is known to be found for regular maintenance of replication forks. Additionally, it is estimated that more than 10^6^ replication-stalling DNA lesions per cell per day form in humans. Because the number of Okazaki fragments per cell cycle is about 20-50 X 10^6^ in humans, one replication fork stalling event may associate with about 20-50 Okazaki fragments theoretically [16,29,63]. This estimate suggests that FEN1 is likely to encounter and interact with FANCA that is recruited to maintain stability of replication forks [44–46]. Our *in vitro* result with the 15-nt 5’ RNA and DNA flaps demonstrated that FANCA is likely to be involved in the removal of RNA/DNA primers by facilitating FEN1 action during maturation of Okazaki fragments. 

FEN1 participates in the long-patch base excision repair of non-bulky DNA lesions (oxidation, methylation, base loss, etc) by interacting with Pol β, APE1, Lig 1, PCNA and Neil1 for efficient removal of the damaged bases and 5’ flaps [17,18,64,65]. FANCA was also shown to be involved in base excision repair through stabilizing the glycosylase Neil1 [66]. Our result indicated that FANCA additionally interacts with FEN1 to facilitate removal of 5’ DNA flap catalyzed by FEN1. Our findings suggest that the Fanconi anemia pathway may directly regulate the excision repair of DNA lesions caused by oxidative stress explaining the oxidative stress sensitive phenotype of Fanconi anemia cells [67].

Because of the preferential binding of FANCA to 5’ flap structures [46], we hypothesized that FANCA may facilitate loading FEN1 to the 5’ flap substrate and therefore increase the substrate affinity of FEN1. However, FANCA increased both the turnover rate and K_m_ of the endonuclease activity of FEN1 (Figure 3). Based on these results, we speculate that FANCA may regulate the endonuclease activity of FEN1 through two possible mechanisms: (i) Direct protein-protein interaction between FANCA and FEN1 that changes FEN1 conformation and increases its endonuclease turnover and (ii) competition for the 5’ flap substrate between FANCA and FEN1 that reduces substrate affinity of FEN1. It is conceivable that reduced substrate affinity of FEN1 in the presence of FANCA may also cause faster release of the incision product and therefore help FEN1 to turnover. Overall, these possible mechanisms result in about two-fold increase in FEN1 enzyme efficiency. This possible mechanism is supported by the data that both non-nucleic-acids-binding N- and nucleic-acids-binding C-terminals of FANCA are required for FEN1 stimulation (Figure 4). It remains to be determined how FANCA exactly interacts with FEN1, but it is distinct from WRN, RFC, and PCNA in interacting with FEN1. Both WRN and RFC increased the turnover of FEN1 without affecting the substrate affinity, on the other hand, PCNA increased the substrate affinity of FEN1 without changing the turnover rate [25,28,57]. 

The next interesting question remaining to be answered is whether FANCA affects other activities of FEN1. Like FANCA, the gap-dependent endonuclease as well as the exonuclease of FEN1 is important for rescue of stalled replication forks [7,47,48]. It has been shown that defects in the gap-dependent endonuclease and exonuclease of FEN1 cause chronic inflammation and cancers [22]. Coincidently, deficiency in FANCA also results in inflammation and cancers [50–52]. Based on the physical and functional interactions between FANCA and FEN1, it is conceivable that FANCA may regulate the gap-dependent endonuclease as well as exonuclease activities of FEN1 and therefore contribute to suppression of inflammatory responses and maintenance of genomic stability.

## Supporting Information

Figure S1 (**A**) SDS-PAGE analysis of purified FANCA and FEN1 proteins. Proteins were subjected to a 10% gel and the gel was stained with Coomassie Brilliant Blue R-250. Protein markers in kilodaltons were indicated. (**B**) Diagrams and sequence sequence of the 15-nt DNA and RNA flaps.
(PDF)

Figure S2 **Kinetic analyses of FEN1 endonuclease activity were performed with or without WT FANCA protein for both ^32^P-labeled 15-nt length DNA and RNA 5’ flap substrates with increasing amount of non-labeled “cold” substrates to a final concentration of 0, 2, 6, 20, 60, 200 nM.** The concentration of ^32^P-labeled “hot” substrates was 1 nM. The concentration of FEN1 was 0.2 nM for DNA flap and 0.1 nM for RNA flap. The concentration of FANCA was 10 nM. Reaction products were resolved in 15% denaturing polyacrylamide gel. Arrows point to the incision products. The incision product band is quantified, converted to the final concentration and shown on the bottom.
(PDF)
