# Supplementary figures and images for: Human Fanconi Anemia Complementation Group A Protein Stimulates the 5’ Flap Endonuclease Activity of FEN1
Source: PLoS One. 2013 Dec 4;8(12):e82666. doi: 10.1371/journal.pone.0082666 (PMC3857783; doi:10.1371/journal.pone.0082666)

A

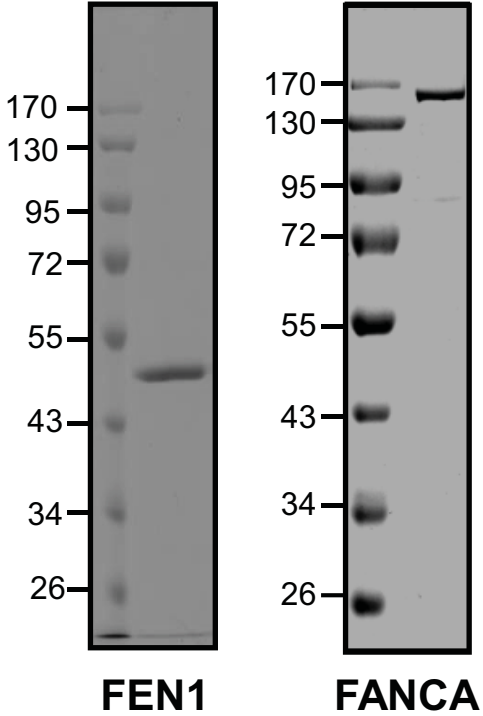

B

15-nt 5' DNA flap:

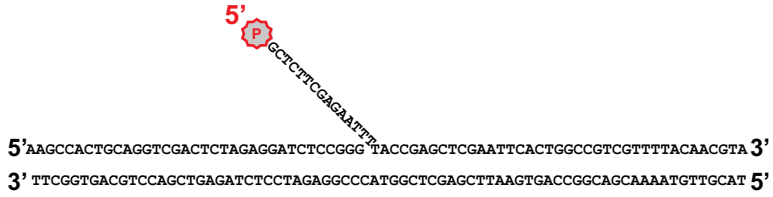

15-nt 5' RNA flap:

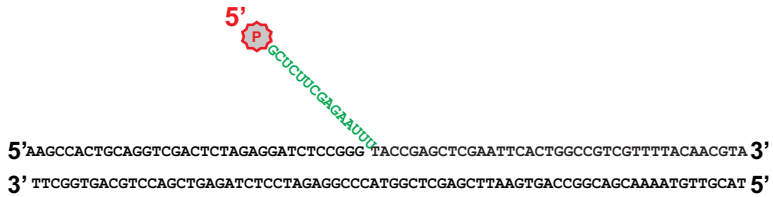

Figure S1

Supplement: Figure S1 — (A) SDS-PAGE analysis of purified FANCA and FEN1 proteins. Proteins were subjected to a 10% gel and the gel was stained with Coomassie Brilliant Blue R-250. Protein markers in kilodaltons were indicated. (B) Diagrams and sequence sequence of the 15-nt DNA and RNA flaps. (PDF) [file pone.0082666.s001.pdf]

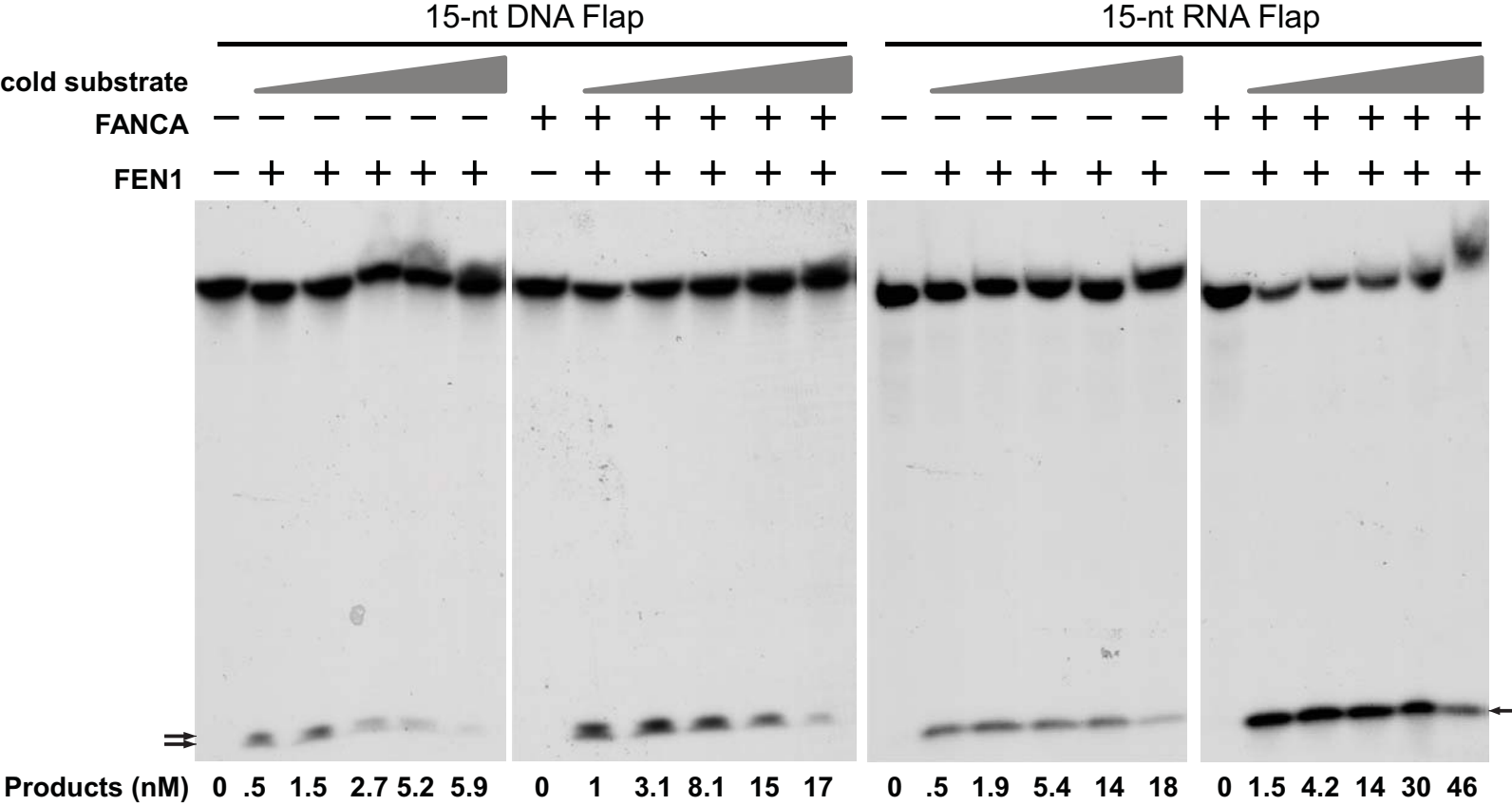

Figure S2

Supplement: Figure S2 — Kinetic analyses of FEN1 endonuclease activity were performed with or without WT FANCA protein for both 32P-labeled 15-nt length DNA and RNA 5’ flap substrates with increasing amount of non-labeled “cold” substrates to a final concentration of 0, 2, 6, 20, 60, 200 nM. The concentration of 32P-labeled “hot” substrates was 1 nM. The concentration of FEN1 was 0.2 nM for DNA flap and 0.1 nM for RNA flap. The concentration of FANCA was 10 nM. Reaction products were resolved in 15% denaturing polyacrylamide gel. Arrows point to the incision products. The incision product band is quantified, converted to the final concentration and shown on the bottom. (PDF) [file pone.0082666.s002.pdf]
